# AMPD1 Is Associated With the Immune Response and Serves as a Prognostic Marker in HER2-Positive Breast Cancer

**DOI:** 10.3389/fonc.2021.749135

**Published:** 2021-11-09

**Authors:** Long Wang, Xue Zhang, Mengxue Wang, Yunhai Li, Jiali Xu, Jiaying Wei, Hongzhong Li, Guosheng Ren, Xuedong Yin

**Affiliations:** ^1^ Department of Endocrine and Breast Surgery, The First Affiliated Hospital of Chongqing Medical University, Chongqing, China; ^2^ Key Laboratory of Molecular Oncology and Epigenetics, The First Affiliated Hospital of Chongqing Medical University, Chongqing, China

**Keywords:** HER2-positive breast cancer, AMPD1, tumor microenvironment, tumor-infiltrating immune cells, immunotherapy

## Abstract

**Background:**

Although immunotherapy has been used in the treatment of metastatic triple negative breast cancer (TNBC), its therapeutic influence on human epidermal growth factor receptor 2 (HER2)-positive subtype remains controversial. It is therefore imperative to find biomarkers that can predict the immune response in HER2+ BC.

**Methods:**

ESTIMATE was utilized to compute the ImmuneScore and StromalScore from data obtained from TCGA database, and differentially expressed genes (DEGs) were identified. In addition, univariate Cox regression was used to assess candidate genes such as AMPD1, CD33, and CCR5. Gene set enrichment analysis (GSEA) was used to further understand AMPD1-associated pathways. Moreover, immunohistochemical analyses were performed to further reveal the relationship among AMPD1, CD4 and CD8 genes.

**Results:**

The expression of AMPD1 was markedly associated with disease outcome and tumor-infiltrating immune cells (TICs). In addition, AMPD1 was associated with lymph node status, age and the expression of PD-L1 and PD-L2. High AMPD1 expression was linked to longer overall survival (OS). Upregulated expression of AMPD1 correlated with the enrichment of immune-related signaling pathways. In addition, immunohistochemical analyses demonstrated a co-expression profile among AMPD1, CD4 and CD8 genes.

**Conclusions:**

Taken together, our data demonstrated that AMPD1 might serve as a novel biomarker for predicting the immune response and disease outcome in HER2+ BC.

## Introduction

Breast cancer (BC) is the most frequently occurring cancer and the primary cause of cancer-associated mortality in women (1, 2). BC is clinically classified into four subclasses: luminal A, luminal B, HER2+ and triple-negative breast cancer (TNBC). Tumor-infiltrating immune cells (TICs) are associated with the tumor response to neoadjuvant chemotherapy; these cells are more frequently detected in the HER2+ BC and TNBC subtypes, and have been found to mediate immunotherapy (3–5). Furthermore, programmed death ligand-1 (PD-L1), a biomarker for immunotherapy response, is more frequently expressed in HER2+ BC and TNBC than in the other two subtypes (6, 7). Immunotherapies such as antagonists to immune checkpoint inhibitors (e.g., PD-L1 and PD-1) have demonstrated remarkable clinical effects against several cancers, including BC (8–10). Recently, the U.S. Food and Drug Administration approved chemoimmunotherapy combinations for treating metastatic non–small cell lung cancer and PD-L1 positive metastatic TNBC (11).

Clinical trials have investigated the use of immunotherapy combined with anti-HER2 targeted therapy in HER2+ BC (12). However, the results from these studies remain controversial. For instance, in a clinical phase 1b-2 trial, pembrolizumab (PD-1 antibodies) combined with trastuzumab in women with trastuzumab-resistant, metastatic HER2+ BC has been shown to be safe and effective. Moreover, no dose-limiting toxicity symptoms were observed (13). In contrast, another study has reported that three of eight patients had dose-limiting toxicity symptoms that required dose-reduction after the first dose, and no objective antitumor responses were observed after dose reduction (14). Therefore, identifying novel prognostic and therapeutic biomarkers that might predict the immune response in the HER2+ BC is important.

Infiltration of immune cells in tumor sites and immune responses are the basis for effective immunotherapy (4, 15, 16). However, immunotherapy has poor efficacy in tumors that lack immune infiltration (17). Therefore, evaluation of factors that might be used to assess the immune response in cancers with abundant immune cell infiltration would support the development of immunotherapy. Here, we analyzed the proportion of TICs and the ratio of immune and stromal components in HER2+ BC samples in The Cancer Genome Atlas (TCGA) data resource. We used the ESTIMATE and CIBERSORT algorithms and identified adenosine monophosphate deaminase 1 (AMPD1) as an immune-associated biomarker in HER2+ BC.

Adenosine monophosphate deaminase 1 (AMPD1) is a deaminase that catalyzes the deamination of adenosine monophosphate (AMP) to inosine monophosphate (IMP) in purine nucleotide metabolism and energy metabolism (18, 19). AMPD1 deficiency in humans is closely associated with exercise-induced myopathy and muscle fatigue (20). However, little research has been performed on AMPD1 in tumors, particularly its relationship with the immune response in HER2+ BC. Our findings showed that AMPD1 may serve as a potential biomarker in predicting the immune response and disease outcome in HER2+ BC.

## Materials and Methods

### Raw Data

We obtained RNA-seq data and the full clinical data from 893 breast cancer cases (non-malignant samples,139 cases; cancer samples, 754 cases) from TCGA database. We then analyzed patients with HER2 expression status (paratumor samples, 23 cases; tumor samples, 176 cases) among the 893 cases in IHC (TCGA: https://cancergenome.nih.gov/). Detailed sample information is given in **Supplementary Tables 1**, **2**.

### Calculation of ImmuneScore, StromalScore, and ESTIMATEScore

We used ESTIMATE to calculate the ImmuneScore, StromalScore and ESTIMATEScore, which represent the immune matrix components of each BC or HER2+ BC sample in the TME and predict the immune status: the higher the score, the greater the corresponding component in the TME. Detailed score information is given in **Supplementary Tables 1**, **2**.

### Analysis of Scores With Clinical Stages

The clinicopathological data matching the HER2+ BC samples were abstracted from TCGA data resource. Data analyses were implemented in the R software. On the basis of the number of clinical stages being compared, Wilcoxon rank sum or Kruskal–Wallis rank sum test was performed. Detailed clinical information is given in **Supplementary Table 2**.

### DEGs Between The High- and Low-Score Groups According to the ImmuneScore and StromalScore

On the basis of the median ImmuneScore or StromalScore, we divided 171 HER2+ BC malignant samples into high- or low-score groups. The limma package was used to assess differential gene expression, and we obtained DEGs through comparing the expression between the high- and low-score samples. The DEGs exhibiting a fold change greater than 1 after log2 transformation (high-score group/low-score group) and an FDR of < 0.05 were regarded as significant. Heatmaps of the DEGs were generated with the pheatmap R package.

### GO, KEGG, and Cox Regression Analysis

The R packages cluster profiler, enrichplot and ggplot2 were used for GO and KEGG analyses. A threshold p-value and q-value was set to 0.05. The R survival package was used to perform univariate Cox regression. The analysis ranked a total of 19 genes. Among 19 candidate genes, the expression differences between the HER2+ BC tumor tissues and paratumor tissues were evaluated with Wilcoxon rank sum test. Details are presented in **Supplementary Figure 1**.

### Gene Set Enrichment Analysis

Hallmark and the Immunologic signature data were abstracted from the Molecular Signatures Data Resource. Afterward, gsea-4.1.0 software was used to perform GSEA. GSEA was conducted on the entire transcriptome data from HER2+ BC malignant samples, with NOM p < 0.05 and FDR q < 0.05 signifying significant DEGs.

### TIC Profile

The CIBERSORT data resource was used to determine the TIC richness profile in 176 HER2+ BC tumor samples. Subsequently, quality filtering was performed, in which samples with p < 0.05 were chosen for downstream analyses. Detailed CIBERSORT results are given in **Supplementary Table 2**.

### Immunohistochemistry

The HER2+ BC and TNBC sample tissues were obtained from the First Affiliated Hospital of Chongqing Medical University (CQMU). Immunohistochemistry and IHC scores were analyzed according to a previously described protocol (21). We used anti-AMPD1 (Proteintech, 19780–1-AP), anti-CD4 (Abcam, ab183685) and anti-CD8α (CST, #70306) as primary antibodies against AMPD1, CD4 and CD8 proteins. Serial sections from the human samples were used to perform the IHC assays. Our HER2+ BC samples and clinical information are given in **Supplementary Table 3**. Representative IHC images of AMPD1 protein expression in two TNBC samples are shown in **Supplementary Figure 2**.

### Ethical Approval

This study was approved by the Institutional Ethics Committees of the First Affiliated Hospital of Chongqing Medical University and was bound by the Declaration of Helsinki. The patients/participants provided written informed consent to participate in this study.

## Results

### Immune and Stromal Components in the TME Correlate With the Prognosis of HER2+ BC

**Figure 1:**
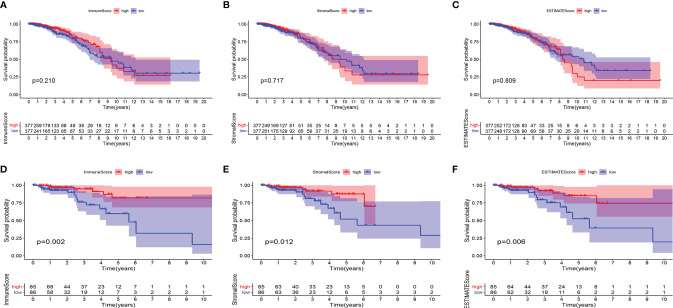
Associations of ImmuneScore, StromalScore, and ESTIMATEScore with survival among BC or HER2+ BC patients. Kaplan–Meier survival analyses of breast cancer patients with low and high **(A)** ImmuneScores (p = 0.210), **(B)** StromalScores (p = 0.717), and **(C)** ESTIMATEScores (p = 0.809). Kaplan–Meier survival analyses of HER2+ BC patients with low and high **(D)** ImmuneScores (p = 0.002), **(E)** StromalScores (p = 0.012), and **(F)** ESTIMATEScores (p = 0.006).

Using the ESTIMATE algorithm, we determined the ratio of the immune matrix components of each sample in the TME *via* the ImmuneScore, StromalScore and ESTIMATEScore. To evaluate the relationship between overall survival (OS) and the levels of stromal invasion, as well as the immune cells, we analyzed the ImmuneScore, StromalScore and ESTIMATEScore with the Kaplan–Meier approach. The three scores showed no clear relationship with OS in patients with BC (**Figures 1A–C**). However, the scores showed a positive relationship with the OS in patients with HER2+ BC (**Figures 1D–F**). The data demonstrated that the immune and stromal components in the TME indicate prognosis in patients with HER2+ BC.

### Invasion of Immune or Stromal Cells Correlates With the Clinicopathological Staging of Patients With HER2+ BC

**Figure 2:**
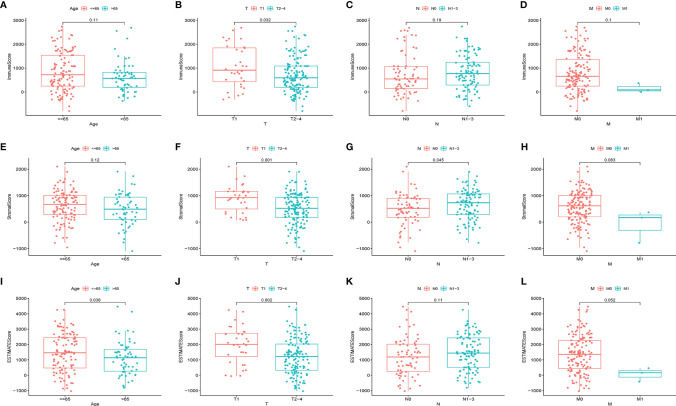
Associations of ImmuneScore, StromalScore, and ESTIMATEScore with age or TNM staging in HER2+ BC patients. Kruskal–Wallis rank sum tests of the associations of **(A–D)** TME immune components, **(E–H)** TME stromal components, and **(I–L)** TME immune and stromal components with age or TNM staging.

The matching clinical data of HER2+ BC were analyzed to elucidate the association of the fraction of immune and stromal constituents with the clinicopathological features from TCGA database. The data showed that the ImmuneScore was markedly associated with T stage (**Figure 2B**), but not with age, N stage or M stage (**Figures 2A, C, D**). Furthermore, the StromalScore was markedly associated with T stage and N stage (**Figures 2F, G**), but not age or M stage (**Figures 2E, H**). In addition, the ESTIMATEScore was markedly associated with age and T stage (**Figures 2I, J**), but not N or M stages (**Figures 2K, L**). Thus, the invasion of immune or stromal cells correlated with tumor development and may serve as a prognostic biomarker in HER2+ BC.

### DEGs in the ImmuneScore and StromalScore Were Markedly Enriched in Immune-Associated Genes

**Figure 3:**
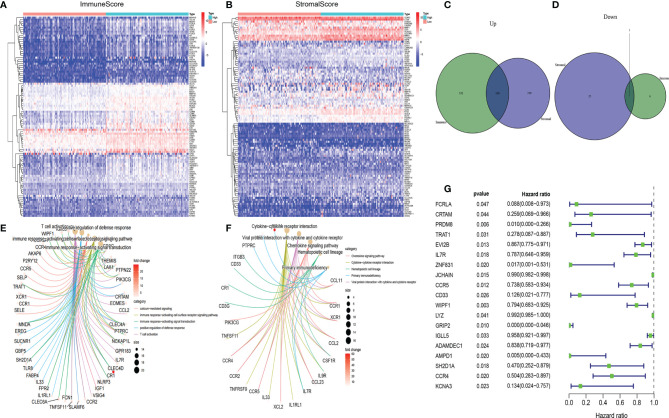
Heatmaps, Venn diagrams, GO, KEGG enrichment and univariate Cox analyses of differentially expressed genes (DEGs) in HER2+ BC. Heatmaps of significant DEGs (FDR-adjusted p < 0.05, |log FC| > 1) between high and low **(A)** ImmuneScore and **(B)** StromalScore groups. Venn diagrams of upregulated and downregulated DEGs shared by the **(C)** ImmuneScore and **(D)** StromalScore analyses. **(E)** GO and **(F)** KEGG enrichment analyses (p < 0.05 and q < 0.05). **(G)** Univariate Cox regression analysis 127 DEGs, showing genes with p < 0.05.

Transcriptome HER2+ BC data from TCGA data resource were analyzed to evaluate the relationship between the gene expression and ImmuneScore or StromalScore. A total of 665 differentially expressed genes (DEGs; 658 upregulated and 7 downregulated) were identified between the high and low ImmuneScore groups, and 531 DEGs (505 upregulated and 26 downregulated) were identified between the high and low StromalScore groups (**Figures 3A, B**). In addition, 127 common DEGs (126 upregulated and 1 downregulated) were found in both analyses (**Figures 3C, D**); these DEGs might be key factors in the TME.We next predicted the roles of the common DEGs with Gene ontology (GO) and Kyoto Encyclopedia of Genes and Genomes (KEGG) analyses. The GO data illustrated that most of the DEGs clustered in the immune-linked GO terms, such as T-cell activation and positive modulation of defense response (**Figure 3E**). The KEGG pathway enrichment analysis demonstrated enrichment of cytokine-cytokine receptor cross-talk and viral protein cross-talk with the cytokine-chemokine signaling cascade (**Figure 3F**). Consequently, the DEGs might be associated with immune activity; thus, immune factors play an essential role in the TME in individuals with HER2+ BC. Among the 127 DEGs, we determined the significant factors by performing univariate Cox regression for the survival of individuals with HER2+ BC. Consequently, we evaluated 19 factors (p < 0.05) (**Figure 3G**). Relative mRNA expression levels of the 19 candidate genes in tumor and paratumor samples were analyzed. Most genes did not show any significant changes (**Supplementary Figure 1**). Therefore, we selected AMPD1 for further study. AMPD1 was observed to be lower in tumor tissues and simultaneously was associated with better prognosis in HER2+ BC patients.

### Correlation of AMPD1 Expression With Survival and TNM Stage in Patients With HER2+ BC

**Figure 4:**
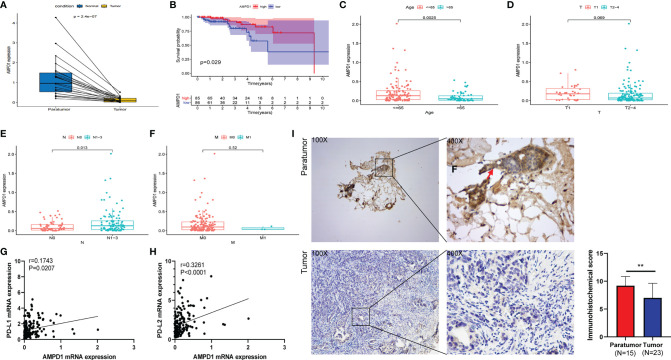
AMPD1 expression, survival, and clinicopathological characteristic analyses. **(A)** AMPD1 was differentially expressed between HER2+ BC tumor tissues and paratumor tissues (Wilcoxon rank sum test p < 0.001). **(B)** Survival analysis of HER2+ BC patients with low and high AMPD1 expression (based on median expression) in the TCGA database (log-rank test p = 0.029). **(C–F)** Kruskal-Wallis rank sum tests of the associations between AMPD1 expression and clinicopathological features. **(G, H)** Correlation analysis of AMPD1 expression and the mRNA levels of PD-L1 (p = 0.0207, rPearson = 0.1743) or PD-L2 (p < 0.0001, rPearson = 0.3261) in the TCGA database. **(I)** Representative IHC images of AMPD1 protein expression in HER2+ BC tumor and paratumor tissues. **p < 0.01.

Our correlation analyses showed downregulation of AMPD1 expression in HER2+ BC tumor tissues, in contrast with the paratumor tissues (p < 0.001) (**Figure 4A**). In addition, the data demonstrated that higher AMPD1 expression predicted better OS in patients with HER2+ BC (**Figure 4B**). Furthermore, AMPD1 expression was associated with age (**Figure 4C**) and lymph node status (**Figure 4E**), but not with tumor size or distant metastasis (**Figures 4D, F**). More importantly, AMPD1 expression was positively correlated with mRNA levels of PD-L1 (Pearson r = 0.1743, p = 0.0207) and PD-L2 (Pearson r = 0.3261, p < 0.0001) (**Figures 4G, H**). To evaluate the expression of AMPD1 protein in clinical biopsy samples, we performed immunohistochemistry, which showed higher expression of AMPD1 in paratumor tissues than in HER2+ BC tissues (**Figure 4I**).

### AMPD1 Expression Indicates TME Perturbation

**Figure 5:**
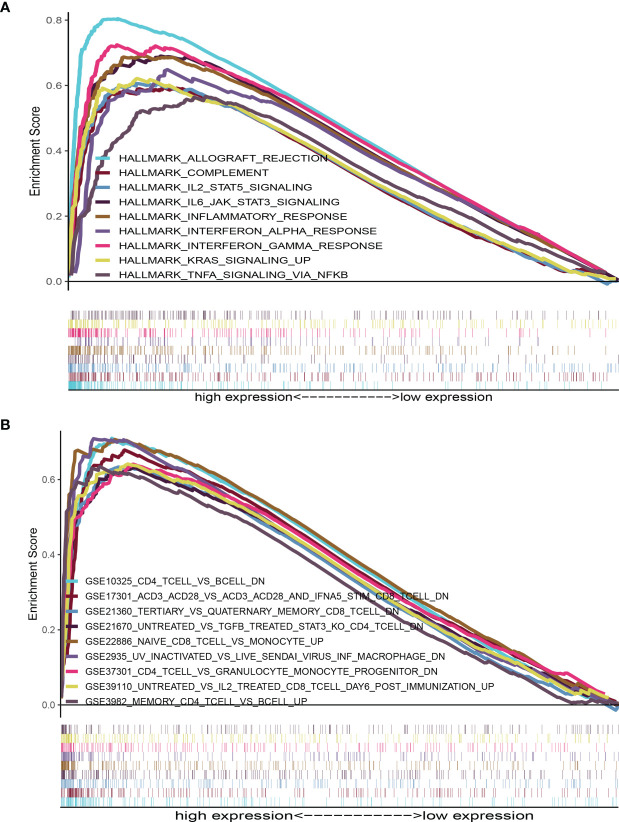
GSEA for HER2+ BC samples with AMPD1 expression. **(A)** The enriched gene sets in HALLMARK collection by the high AMPD1 expression sample (nominal p < 0.05 and FDR-adjusted q < 0.05). **(B)** Enriched gene sets in C7 collection, the immunologic gene sets, by samples with high AMPD1 expression (nominal p < 0.05 and FDR-adjusted q < 0.05).

HALLMARK and C7 collection were evaluated with GSEA v4.1.0 software. The HALLMARK data illustrated that immune-linked signaling cascades consisting of TNFA signaling, interleukin family signaling and interferon response were abundant in the group with high expression of AMPD1 (**Figure 5A**). For C7 collection, abundant immune functional gene sets were observed in the group with upregulated AMPD1 expression (**Figure 5B**). These data indicated that AMPD1 might be an important marker of the TME immune response in HER2+ BC.

### AMPD1 Expression Correlates With the TIC Fraction

**Figure 6:**
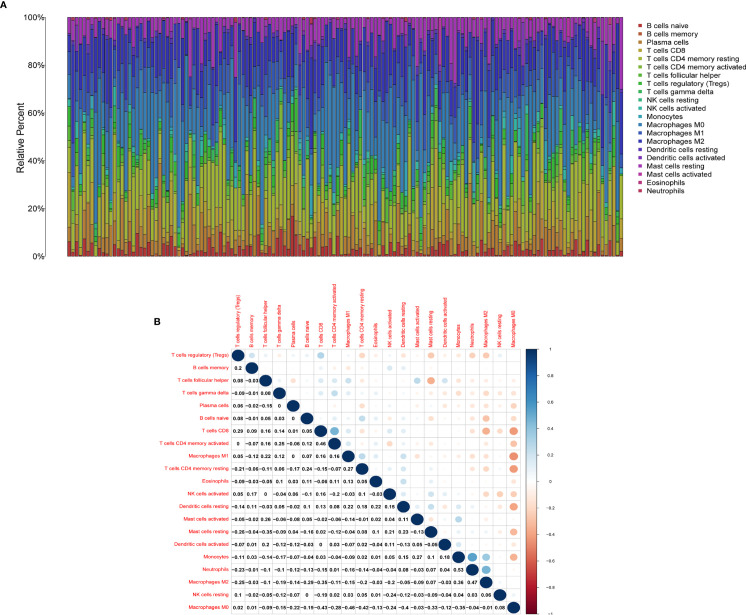
TIC profile in tumor samples and correlation analysis. **(A)** Barplot showing the proportion of 21 kinds of TICs in HER2+ BC samples. **(B)** Heatmap showing the correlation between 21 kinds of TICs and numerals in each small box indicating the p value of correlation between two kinds of cells. The shade of each small color box represents the corresponding correlation value between two cells, and the Pearson coefficient was used for significance testing.

**Figure 7:**
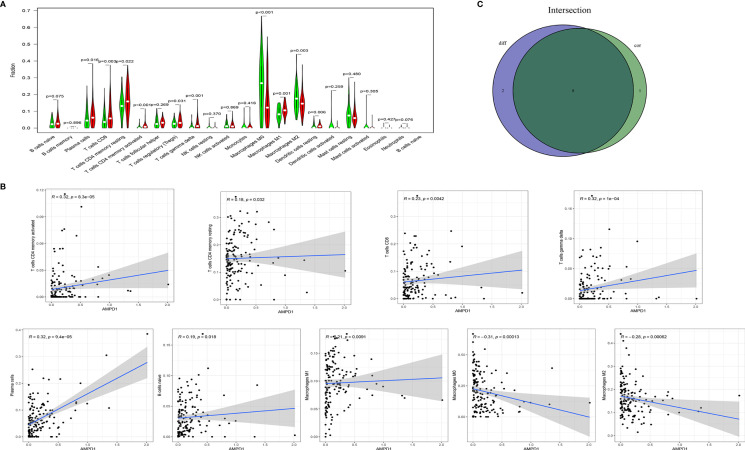
Differences in proportions of TICs between high and low AMPD1 expression groups and correlations of TICs with AMPD1 expression. **(A)** Violin plots of the proportions of 21 immune cell types in tumor tissues with low (green) or high (red) AMPD1 expression, compared using the Wilcoxon rank sum test. **(B)** Scatter plots showing Pearson correlation between the proportions of the 9 TICs and AMPD1 expression. Blue lines denote the best-fit linear models. **(C)** Venn diagram of intersection between analyses in **(A, B)** showing that 8 TICs were shared between the analyses.

**Figure 8:**
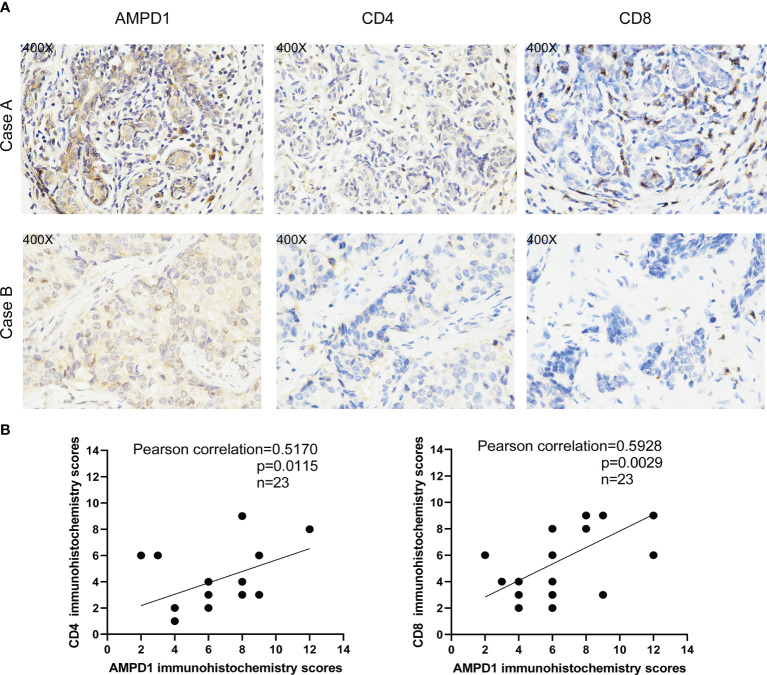
AMPD1 expression is positively correlated with CD4 and CD8 in HER2+ BC tissues. **(A)** Representative images of AMPD1, CD4 and CD8 staining. Case A: High expression of AMPD1, CD4 and CD8; Case B: Low expression of AMPD1, CD4 and CD8. **(B)** The correlations between AMPD1, CD4 and CD8 expression were analyzed by Pearson correlation tests.

We used the CIBERSORT algorithm to evaluate the components of tumor-infiltrating immune cell subsets in each sample of HER2+ BC, which provided the foundation for further correlation analysis of AMPD1 expression with the immune microenvironment (**Figure 6**). The data showed an association between eight TICs and the mRNA levels of AMPD1 (**Figure 7**). In addition, six of the 21 TICs were positively associated with AMPD1 expression: plasma cells, CD4 memory activated T cells, CD4 memory resting T cells, CD8 T cells, gamma delta T cells and M1 macrophages. In contrast, M2 macrophages and M0 macrophages were inversely associated with the AMPD1 expression. Furthermore, our immunohistochemical analyses showed co-expression of AMPD1, CD4 and CD8 in HER2+ BC (**Figure 8**). Thus, we speculated that the expression of AMPD1 might potentially affect the immune response in the TME.

## Discussion

Conflicting findings have been reported regarding the therapeutic effect of immunotherapy on HER2+ BC. Herein, we evaluated the TME-associated genes potentially mediating the immune response and predicting survival in patients with HER2+ BC. Using TCGA datasets, we conducted univariate Cox regression and identified 19 genes including CD33, IL7R and CCR5. Previous studies have associated these three genes with the PD-1/PL-L1 signaling pathway and T cell-mediated immune responses (22–24). Among these genes, AMPD1 was significantly expressed and was associated with disease outcome and the TICs that affect the immune response. Interestingly, the data showed that AMPD1 might be involved in immune activities, and positively correlate with the expression of PD-L1 and PD-L2 in HER2+ BC.

The TME consists of tumor cells, stromal cells and immune cells, which are major mediators in the development of malignancy (25, 26). Recent research has illustrated that low immune cell invasion remains a major obstacle for cancer immunotherapy (27, 28). In addition, the ImmuneScore has been shown to be a prognostic tool for quantifying immune cell infiltrates in situ (29). Our results based on TCGA transcriptome data revealed that the ImmuneScore, StromalScore and ESTIMATEScore have a direct relationship with OS in patients with HER2+ BC, thus suggesting that TME-associated factors may potentially be used to estimate prognosis (30, 31).

Therefore, *via* the ImmuneScore and StromalScore, we selected TME-associated DEGs. GO and KEGG analyses illustrated that most of the selected DEGs participate in immune-linked processes. Further analysis demonstrated that AMPD1 expression was markedly associated with clinic-pathological features and prognosis. Moreover, adenosine plays crucial roles in the establishment of an immunosuppressive TME (32). AMPD1 catalyzes the conversion of adenosine monophosphate to inosine monophosphate (33) and thus might be involved in the regulation of immune responses. In addition, a recent report has demonstrated that AMPD1 expression in the serum of individuals with PTC is downregulated and mediates the development of PTC; these findings may guide diagnosis and the treatment in clinical practice (34). Our data indicated that, compared with the paratumor tissues, HER2+ BC samples showed suppression of AMPD1 expression. High AMPD1 expression was associated with better prognosis, in agreement with findings from a previous bioinformatics analysis study (35). Here, we showed that AMPD1 might participate in the immune response in HER2+ BC, thus providing new ideas for the treatment of HER2+ BC. However, more investigations are needed to define the mechanisms of AMPD1-induced immune activity to better understand the tumor immune microenvironment in HER2+ BC.

The correlation between AMPD1 expression and clinicopathological characteristics showed that AMPD1 might be associated with age and lymph node status. A previous report has shown that the AMPD1 expression in the serum from individuals with PTC is markedly different from the clinicopathological characteristics, such as tumor diameter and TNM stage (34). Interestingly, further analysis indicated that PD-L1 and PD-L2 have a positive relationship with AMPD1 expression. Targeting the PD-1/PD-L1 cascade exhibits remarkable responses to immune checkpoint repression in diverse types of cancers (36–39). Moreover, PD-L2, a relatively less explored ligand of PD-1, is found primarily on activated dendritic cells as well as macrophages, which play an indispensable role in repressing anticancer immune response (40). Therefore, the positive correlation between AMPD1 and PD-L1/PD-L2 expression might provide new ideas for HER2+ BC immune checkpoint therapy.

Furthermore, we performed gene set enrichment analysis (GSEA) and established that immune-linked signaling cascades, consisting of TNFA signaling, interleukin family signaling and interferon response, were markedly enriched in the group with high AMPD1 gene expression. Other studies have demonstrated that the dysregulation of AMPD1 might consequently perturb purine metabolism and guanine and hypoxanthine, thus further affecting the progression of BC (35). The CIBERSORT algorithm was used to identify the fractions of 21 immune cell types in HER2+ BC. AMPD1 expression was positively associated with six cell types (CD4+ memory resting T cells, M1 macrophages, plasma cells, CD4+ memory activated T cells, gamma delta T cells and CD8+ T cells) and inversely associated with two cell types (M0 macrophages and M2 macrophages). Furthermore, previous investigations have shown that elevated invasion of distinct immunocytes consisting of CD8 T cells, M1 macrophages, resting memory CD4 cells and activated memory CD4 cells is associated with better prognosis (41–43). In contrast, higher numbers of M0 macrophages, NK cells and M2 macrophages reflect dismal clinical outcomes (44–46). Our results demonstrate that AMPD1 might participate in the regulation of immune cell development and function. In addition, immunohistochemical analyses showed that the expression of AMPD1 is suppressed in cancerous tissues. Finally, AMPD1 is co-expressed with CD4 and CD8 in HER2+ BC.

Although PD-1/L1 inhibitors have demonstrated remarkable clinical effects against PD-L1 positive TNBC, this strategy is currently relevant only to a minority of patients with breast cancer (47–49). Moreover, clinical trials have investigated the use of immunotherapy combined with anti-HER2 targeted therapy in HER2+ BC. However, controversies in this field remain (12–14). Our data illustrate that upregulating AMPD1 expression in HER2+ BC tissues might affect the immune response according to the correlation of AMPD1 with TIC profiles or PD-L1 or PD-L2. Hence, AMPD1 may serve as an immune-associated biomarker in the management of HER2+ BC.

## Data Availability Statement

The original contributions presented in the study are included in the article/**Supplementary Material**. Further inquiries can be directed to the corresponding authors.

## Ethics Statement

This research was approved by the Institutional Ethics Committees of the First Afliated Hospital of Chongqing Medical University (#2017–012) and conformed to the tenets of the Declaration of Helsinki.

## Author Contributions

Conceived and designed the experiments: HL, XY, and GR. Performed data collection: LW, XZ, and MW. Analyzed the data: LW, JW and YL. Contributed reagents, materials, or analysis tools: LW, and XZ. Contributed to the writing of the manuscript: LW, MW, and JX. All authors reviewed the manuscript.

## Funding

This study was supported by National Natural Science Foundation grants (#81402178, #81372238).

## Conflict of Interest

The authors declare that the research was conducted in the absence of any commercial or financial relationships that could be construed as a potential conflict of interest.

## Publisher’s Note

All claims expressed in this article are solely those of the authors and do not necessarily represent those of their affiliated organizations, or those of the publisher, the editors and the reviewers. Any product that may be evaluated in this article, or claim that may be made by its manufacturer, is not guaranteed or endorsed by the publisher.
