# Supplementary material for: AMPD1 Is Associated With the Immune Response and Serves as a Prognostic Marker in HER2-Positive Breast Cancer
Source: Front Oncol. 2021 Nov 9;11:749135. doi: 10.3389/fonc.2021.749135 (PMC8660114; doi:10.3389/fonc.2021.749135)
Supplement: Supplementary file 1 [file DataSheet_1.pdf]

frontiers

Supplementary Material

1 Supplementary Figures and Tables

1.1 Supplementary Figures

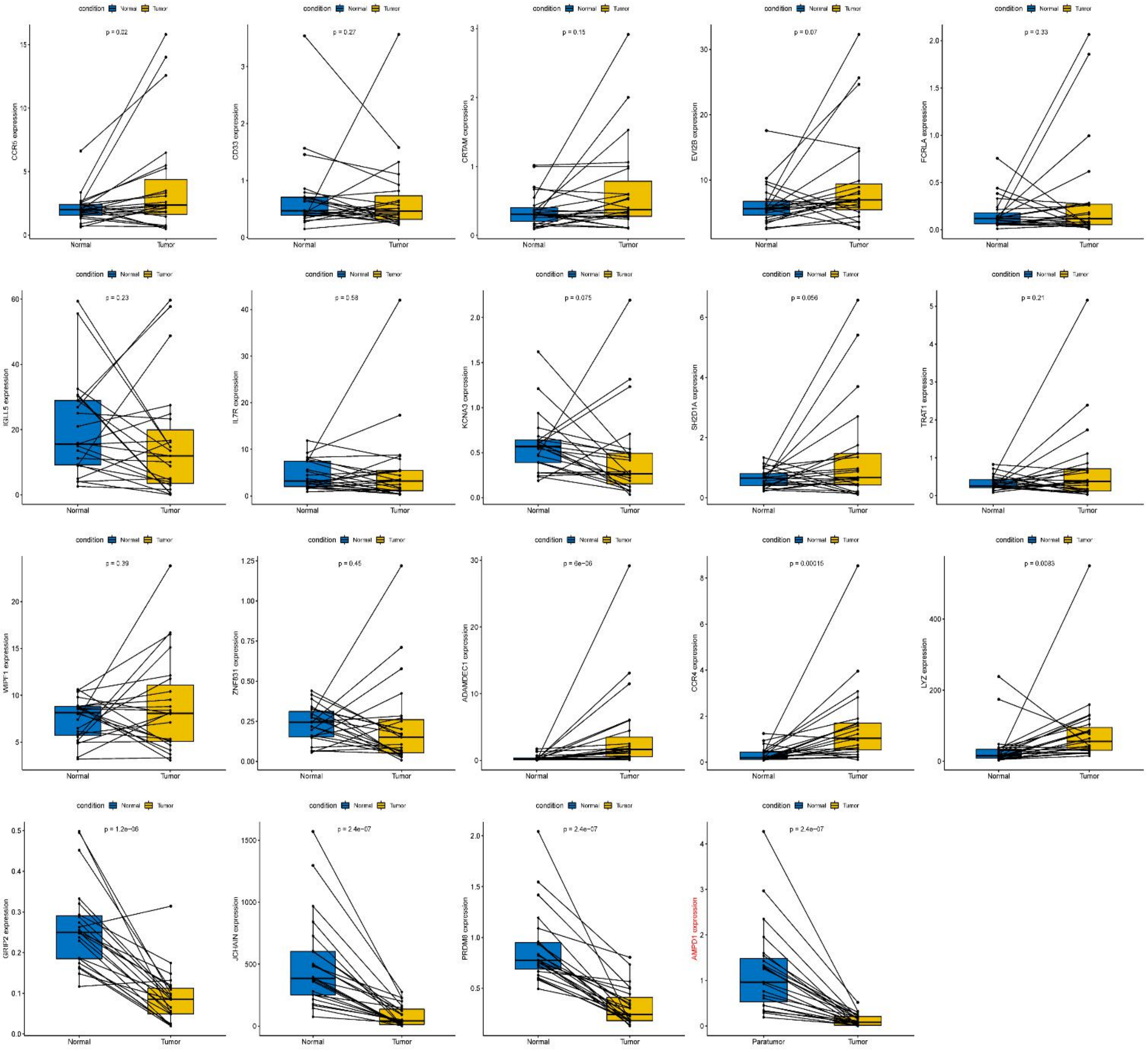

Supplementary Figure 1. Relative mRNA expression levels of the 19 candidate genes in HER2-positive breast cancer tumor and paratumor samples from TCGA database were analyzed. Most genes did not show any significant changes. Thus, AMPD1 was selected for further study.

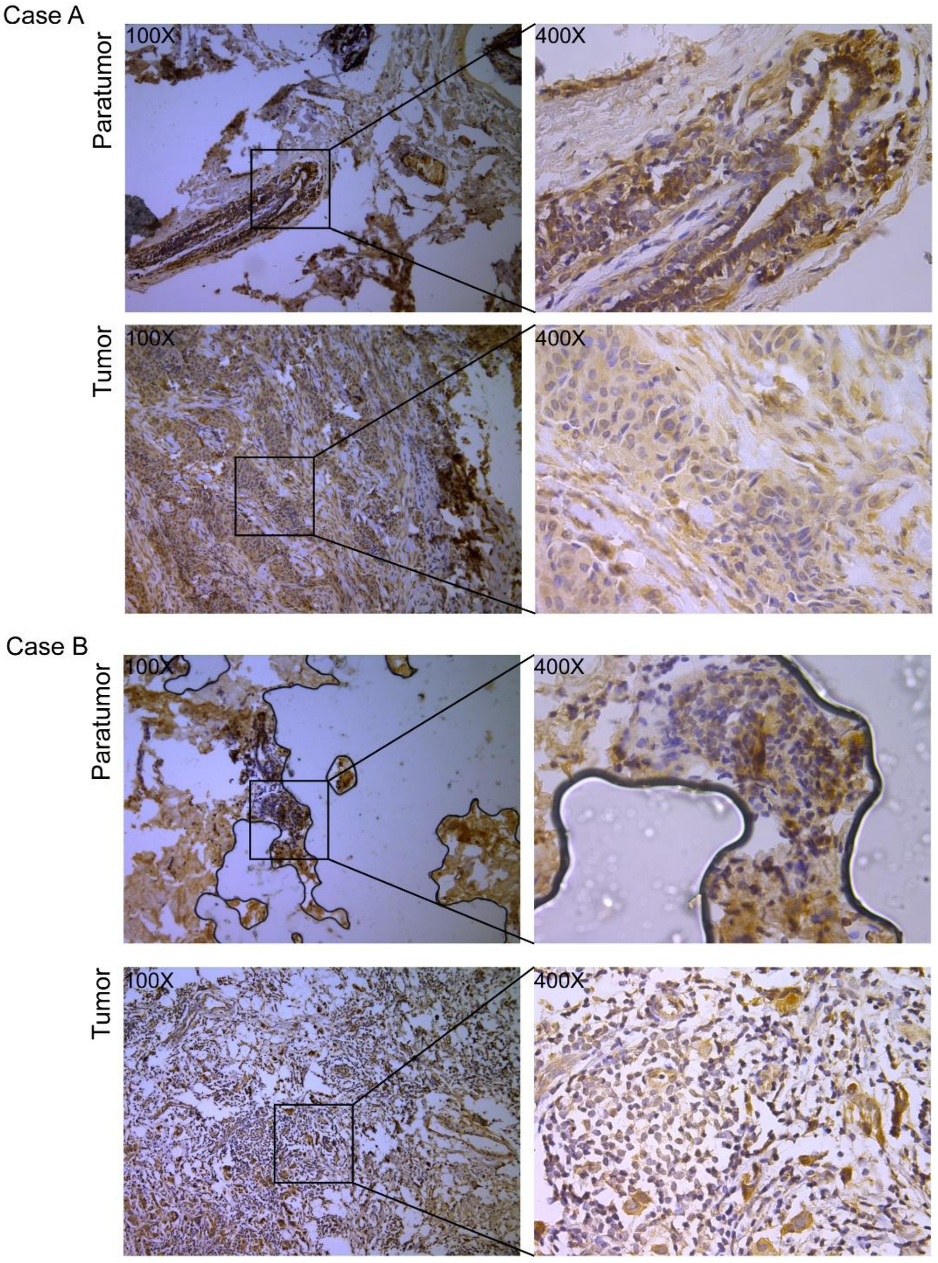

Supplementary Figure 2. Representative IHC images of AMPD1 protein expression in two triple-negative breast cancer (TNBC) samples from our laboratory biobank.

1.2 Supplementary Tabels

Supplementary Tabel 1. The clinical and samples information of Breast cancer from TCGA database.

Supplementary Tabel 2. The clinical and samples information of HER2-positive breast cancer from TCGA database.

Supplementary Tabel 3. The AMPD1 IHC scores and clinical information of HER2-positive breast cancer from our laboratory biobank.
